# Genotype Characterization of Commonly Used Newcastle Disease Virus Vaccine Strains of India

**DOI:** 10.1371/journal.pone.0098869

**Published:** 2014-06-04

**Authors:** Sohini Dey, Madhan Mohan Chellappa, Satish Gaikwad, Jag Mohan Kataria, Vikram N. Vakharia

**Affiliations:** 1 Division of Veterinary Biotechnology, Indian Veterinary Research Institute, Izatnagar, Bareilly, India; 2 OIE Reference Laboratory for Newcastle Disease, Avian Diseases Section, Animal and Plant Quarantine Agency, Anyang, South Korea; 3 Central Avian Research Institute, Izatnagar, Bareilly, India; 4 Institute of Marine and Environmental Technology, University of Maryland, Baltimore County, Baltimore, Maryland, United States of America; Virginia Polytechnic Institute and State University, United States of America

## Abstract

Newcastle disease is an avian pathogen causing severe economic losses to the Indian poultry industry due to recurring outbreaks in vaccinated and unvaccinated flocks. India being an endemic country, advocates vaccination against the virus using lentogenic and mesogenic strains. Two virus strains which are commonly used for vaccination are strain F (a lentogenic virus) and strain R2B (a mesogenic virus). Strain F is given to 0–7 days old chicks and R2B is given to older birds which are around 6–8 weeks old. To understand the genetic makeup of these two strains, a complete genome study and phylogenetic analysis of the F, HN genes of these vaccine strains were carried out. Both the viral strains had a genome length of 15,186 nucleotides and consisted of six genes with conserved complimentary 3' leader and 5' trailer regions. The fusion protein cleavage site of strain F is GGRQGRL and strain R2B is RRQKRF. Although both the viral strains had different virulence attributes, the length of the HN protein was similar with 577 amino acids. Phylogenetic analysis of F, HN and complete genome sequences grouped these two strains in genotype II category which are considered as early genotypes and corroborated with their years of isolation.

## Introduction

Newcastle disease virus (NDV), the prototype of paramyxovirus, causes the highly contagious Newcastle disease (ND) in many avian species, resulting in substantial economic losses in the poultry industry worldwide. Strains of NDV are classified into three main pathogens as highly virulent (velogenic), intermediate (mesogenic) and non-virulent (lentogenic) on the basis of their pathogenicity for chickens. NDV is a member of the Avulavirus genus in the Paramyxovirus family [1]. NDV genome is approximately 15 kb long, non-segmented, single-stranded, negative-sense RNA that codes for six proteins: nucleoprotein (NP), phosphoprotein (P), matrix (M) protein, fusion (F) protein, haemagglutinin-neuraminidase (HN) protein, and polymerase (L) protein [2]. Although NDV is monotypic in nature, both antigenic and genetic diversities are recognised among NDV isolates. Two different systems classifying NDV are currently used worldwide. One of the systems classifies NDV into two major divisions represented by Class I and Class II, with Class I being further divided into nine genotypes and Class II into ten, when comparing the sequences isolated [3], [4], [5]. Class II viruses have been studied in more detail and the genotypes that are considered ‘early’ (1930–1960) I, II, III, IV and IX contain 15,186 nucleotides [4]. Viruses that emerged ‘late’ (after 1960) V, VI, VII, VIII and X contain 15,192 nucleotides. Vaccination of commercially reared birds is the only way to reduce disease and the losses resulting from infection. India, being an endemic country for NDV, outbreaks still occurs in spite of regular vaccination programmes. Though many reasons could be attributed to this scenario, presence of the etiological agent in the vicinity may always pose a severe threat even to vaccinated population. This gains importance by the fact that many of the free-roaming local birds, water fowls and wild birds are reported to harbour velogenic NDV without manifesting clinical signs [6].

Currently, lentogenic NDV strains Hitchner B1, La Sota, Fuller (F), and mesogenic strain R2B are widely used as live vaccines in India. Strain F NDV is a virus of low virulence originally reported by Asplin [7] in England. Since then, in several countries in Europe, Africa and Asia, the use of the virus as an immunizing agent in the form of a live vaccine has been studied [8]. Similarly the vaccine strain R2B used in the Indian subcontinent has given excellent results in older birds (>6 to 8 weeks old) with long lasting immunity but has been proven to be severely pathogenic for baby chicks. The virus strain had originated by passaging three Indian field isolates in embryonated chicken eggs, with one of the lines showing signs of attenuation after 19 passages [9]. Recently the complete genome sequence of NDV mesogenic strain R2B was elucidated [10]. In this paper, we elucidate the complete genome of the hitherto lentogenic NDV strain ‘F’ and studied the genotype characterization of the commonly used vaccine strains in India, namely ‘F’ and ‘R2B’.

## Materials and Methods

### Virus strains

NDV strain F (referred to as Dobson's ‘F’ strain) seed virus was obtained from the viral repository maintained at the Indian Veterinary Research Institute. This virus had undergone 8 serial passages in embryonated eggs in April 1953 when it was received at this Institute. It was further propagated to 39 serial passages in embryonated eggs and the seed virus was prepared [11]. The R2B strain of NDV was also obtained from the viral repository at the Indian Veterinary Research Institute. Both the seed viruses were plaque purified for further work.

### Virus propagation, RNA isolation and genome sequencing

The virus was propagated in eleven days-old embryonated chicken eggs and purified according to previously established procedures [12]. Total viral RNA was extracted using Trizol (Sigma, USA), according to manufacturer's instructions. Reverse transcription was carried out using the Thermoscript RT kit (Invitrogen, USA) to synthesize the first strand cDNA. Oligonucleotide primers were synthesized for amplifying the entire genome of F and R2B viral strains as overlapping fragments. The sequences of primers used in the study are given in Table 1. The leader and trailer regions were generated by rapid amplification of cDNA end (RACE) protocol, as described elsewhere [13]. Three recombinant plasmids containing the amplified product from each overlapping fragment were purified and sequenced in ABI 3730 DNA analyzer (Applied Biosystems, USA) at the DNA sequencing facility, University of Delhi, South Campus, New Delhi.

**Table 1:** Primers used in this study to amplify R2B and F strains of NDV.

Name	Primer sequence (5′–3′)	Genomic location^ab^	Expected size
**Strain R2B**			
1F^c^	ACCAAACAGAGAATCCGTGAA	1–21	1900 bp
1R^d^	GTGAAGGTGGCCATCTTCACT	1880–1900	
2F	ACAACACGGGCACAACTCGAC	1741–1761	1559 bp
2R	GATGAGTCCATCCTGGCACAA	3280–3300	
3F	TTCCATCCCACTGAATGATCG	3191–3211	2171 bp
3R	AGGGTTACCGGTGATCAAGCC	5342–5362	
4F	GGCTCTTTACAATCTGGCTGG	5251–5271	2230 bp
4R	GACTTAGCCATCCGAATCTGG	7461–7481	
5F	GGTTGCGATTGCGTGAGTCAC	7356–7376	2044 bp
5R	GGTTGCGATTGCGTGAGTCAC	9380–9400	
6F	CTAATGGAGGGATTCGCATAC	9242–9262	2046 bp
6R	CCCAATCTCCATTTCCAGGCG	11268–11288	
7F	GTGCACTCATATGTCCTGACC	11093–11113	1951 bp
7R	CGTCGAGTGCAAGAGACTAGC	13024–13044	
8F	CACCCTGTCATCCATTCAAGG	12923–12943	2263 bp
8R	CCCACCAAACAAAGATTTGGTGAATG	15164–15186	
**Strain F**			
1F^c^	ACCAAACAGAGAATCCGTAAGTTA	1–24	2363 bp
1R^d^	GTTTCCGCGGCTGGGTTGACTCCCCT	2338–2363	
2F	AGTCAACCCAGCCGCGGAAACAG	2343–2365	2621 bp
2R	GAGCTGCGGCCGCTGTTATTTG	4943–4964	
3F	AACAGCGGCCGCAGCTCTGAT	4948–4968	1353 bp
3R	TACAACGCGTAGTTTTTTCTTAACTC	6276–6301	
4F	AACTACGCGTTGTAGATGACCAAAG	6288–6312	2078 bp
4R	GCACTACGTATTTTGCCTTGTATCTC	8351–8366	
5F	CAAAATACGTAATGGTAAATAATACGGGTAGGACATG	8358–8383	1694 bp
5R	TCAGCTTAGCGAAGATCCGTCCATTAACT	10024–10052	
6F	CTTCGCTAAGCTGACAAAGAAGTTAAGGAACTG	10039–10071	2805 bp
6R	GTCTAGGCCTCTTACTCTCAGGTAATAG	12817–12844	
7F	AGAGGCCTAGACAATATTGTCT	12833–12854	2353 bp
7R	ACCAAACAAAGATTTGGTGAATGACGAG	15159–15186	

a Genomic location of strain R2B is based on the complete genomic sequence of NDV strain Mukteswar.

b Genomic location of strain F is based on the complete genomic sequence of NDV strain La Sota.

c F stands for forward primer.

d R stands for reverse primer.

### Sequence and phylogenetic analysis

The complete genome sequence analysis of NDV vaccine strains ‘F’ and ‘R2B’ were carried out using the Seqman, Editseq and Megalign modules of the Lasergene software package. Sequences representing genotypes of NDV were aligned using Muscle [14] with default settings implemented in MEGA 5. MEGA 5 was used to select best fit model of nucleotide substitution and phylogenetic analysis was conducted using the nucleotide substitution model under maximum likelihood with boot strap values for 1000 replicates. Markov Chain Monte Carlo method implemented in BEAST was used to generate Bayesian evolutionary analysis. Three independent Markov Chains were run for 70 million generations for complete genome and 10 million for individual genes datasets and first 10% of samples were discarded as burn in. Logs were combined using Logcombiner V 1.7.5. Stationarity was assessed as effective sample size >200 using Tracer Maximum clade credibility tree generated using Tree Annotator V.1.7.5. The support for nodes in Bayesian tree deduction was assessed using posterior probabilities values calculated in BEAST. Trees were visualized using FigTree V 1.4.0. The assignment of genotypes to the sequences used in this study was in accordance to the criteria described by Diel et al. [15].

### Pathogenicity test

NDV vaccine strains F and R2B were subjected to pathogenicity tests that included mean death time (MDT) analysis in 9 days-old specific pathogen free (SPF) embryonated chicken eggs, intra-cerebral pathogenicity index (ICPI) test in one-day old SPF chickens and intra-venous pathogenicity index (IVPI) test in six-weeks old SPF chickens using standard procedures [16].

## Results

### Complete sequences analysis of NDV strains F and R2B

On alignment of the various overlapping fragments generated in this study, it was found that the complete length of the genome of both the vaccine strains of NDV, namely strains ‘F’ and ‘R2B’, is 15,186 nucleotides (nts), which is considered to be of early genotypes [4]. The complete sequence of strain F and strain R2B is available in the GenBank under the accession numbers KC987036.1 and JX316216.1 respectively. The various defining features of the genome of these viral strains are given in Table 2. The 3′ leader sequence of strains F and R2B consists of 55 nts, a length present in all NDV strains [17]. The 5′ trailer sequence of both the strains was 114 nts. It was also found that the 3′- and 5′- termini were highly conserved; especially the first 12 nts in the 3′-terminus and 8 nts in the 5′-terminus were identical. The P gene contains a putative editing site ^477^AAAAAGGG^484^ (mRNA sense) that is identical in position to other NDV strains. The gene order of 3′-N-P-M-F-HN-L-5′ coding from six open reading frames was also similar to other NDV strains.

**Table 2:** Genomic features and protein characteristics of NDV strains R2B and F.

Gene	Hexamer Phasing Position	Gene Length	ORF	IGS	5'UTR	3'UTR	Amino acids	MW (kDa)	pI
**Strain R2B**									
N	2	1746	1470	2	66	210	490	53.2	5.51
P	4	1451	1188	1	83	180	396	41.84	6.21
P/V	4	1452	720	-	83	649	240	25.25	5.63
P/W	4	1453	684	-	83	686	228	24.49	10.11
M	4	1241	1095	1	34	112	365	39.63	9.64
F	4	1792	1662	31	46	84	554	58.8	8.56
HN	3	2002	1734	47	91	177	578	63.17	7.88
L	6	6703	6615	-	11	77	2205	248.44	6.53
**Strain F**									
N	2	1746	1470	2	66	210	490	53.27	5.35
P	4	1451	1188	1	83	180	396	42.38	6.19
P/V	4	1452	720	-	83	649	240	25.53	5.23
P/W	4	1453	546	-	83	824	182	19.48	9.07
M	4	1241	1095	1	34	112	365	39.49	9.59
F	4	1792	1662	31	46	84	554	58.81	8.17
HN	3	2002	1734	47	91	177	578	63.18	7.55
L	6	6703	6615	-	11	77	2205	248.4	7.05

A comparison of NP gene of F and R2B with other vaccine strains of genotype II viruses showed that the amino acid sequence of NP was conserved (the divergence between representative genotype II strains is 0.052±0.004 S.E.) while the C-terminal sequence of NP was relatively variable compared to other regions of NP protein. One of the characteristics of M protein of NDV is nuclear localization, and it is provided by the nuclear localization signals which are due to clusters of basic amino acids in the M protein. There are two highly basic clusters in the position of amino acid sequence from 246 to 263 [18]. The amino acid sequence of both F and R2B, with respect to M protein, is ^246^DRKGKKVTFDKLEKKIR^263^.

Transcription starts in the 3′ leader region, and the genes are transcribed into separate mRNAs in a start-stop-restart mechanism mediated by the conserved transcriptional control sequences at the beginning (Gene Start, GS) and end (Gene end, GE) of each gene. As in other NDV strains, the non-coding intergenic sequences (IGS) lie between GE and GS. The GS, GE and IGS of strains F and R2B for NP, P, M, F, HN and L genes are given in Table 3.

**Table 3:** Sequences of gene start, gene end and intergenic regions of NDV strains R2B and F.

Gene	Gene start sequence	Start position (nt)	Gene end sequence	Intergenic sequences
**Strain R2B**				
**NP**	ACGGGTAGAA	56	TTAGAAAAAA	AT
**P**	ACGGGTAGAA	1804	TAAGAAAAAA	T
**M**	ACGGGTAGAA	3256	TTAGAAAAAA	C
**F**	ACGGGTAGAA	4498	TAAGAAAAAA	CTACCGGATGTAGGTGAACAAAGGCAATAT
**HN**	ACGGGTAGAA	6321	TAAGAAAAAA	TGTAAGTGGCAATGAGATACAAGGCAAAACAGCTCATGGTAAATAGT
**L**	ACGGGTAGGA	8370	TTAGAAAAAA	-
**Strain F**				
**NP**	ACGGGTAGAA	56	TTAGAAAAAA	GT
**P**	ACGGGTAGAA	1804	TAAGAAAAAA	T
**M**	ACGGGTAGAA	3256	TTAGAAAAAA	C
**F**	ACGGGTAGAA	4498	TTAGAAAAAA	CTACGCGTTGTAGATGACCAAAGGACGATAT
**HN**	ACGGGTAGAA	6321	TAAGAAAAAA	TGTAAGTGGCAATGAGATACAAGGCAAAATACGTAATGGTAAATAAT
**L**	ACGGGTAGGA	8370	TTAGAAAAAA	-

### F and HN gene analysis

The F protein is considered as a key determinant in virulence of NDV [19]. However, recent analysis of the complete genome sequences of viral isolates and by reverse genetics, it has been proven that the fusion protein cleavage site (FPCS), spanning amino acids 112 to 117, of the F protein alone cannot confer virulence to an otherwise avirulent strain [20], [21]. In this context, the FPCS of strain F is GGRQG
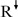
L and that of R2B is RRQK
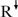
F. Virulent NDV strains typically contain a polybasic cleavage site (R-X-K/R-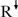
F), which is recognized by most cells. In addition, the presence of phenylalanine (F) residue at position 117 has been described as being possible contributor to neurological effects [22]. In contrast, the lentogenic strains are being encoded by a single pair of basic amino acids and F at position 117 being replaced by L. This further substantiates the fact that strain F is lentogenic while R2B is mesogenic.

HN is a type II homotetrameric glycoprotein with a monomer length of 577 amino acids for most NDV strains [23]. The ability to bind a sialic acid containing receptor is one of the functions of the HN protein and plays a key role in the initial steps of the NDV life cycle [24]. Several amino acid residues, R 174, Y 526, E 401 and R 416 in HN protein were reported to be involved in the activity of sialic acid binding [25]. The salient features of HN protein of strains F and R2B include the following conserved amino acids: R 174, I 175, D 198, K 236, R 416, R 498, Y 526 and E 547; potential glycosylation sites being G_1_-119, G_2_-341, G_3_-433, G_4_-481 and G_6_-538, which is similar to genotype II vaccine strains Beaudette C, B1 and Ulster. The glycosylation site G_5_ present in other NDV strains has been replaced by serine (G_5_-N508S). Further, there is a conserved amino acid E 347 present both in F and R2B, a feature common to all vaccine strains of genotype II ND viruses. The percent similarity in relation to amino acids between F and R2B is 91% and that of R2B and Mukteswar is 96%. The most variable portion of NDV HN protein is present in the N-terminal 78 amino acids that included the transmembrane domain. The predicted amino acids present in the transmembrane domain of strain R2B and Mukteswar is ^27^IAALLLMVITLAVSAVALAYSME^49^, which is different from that of strain F –^27^IAILLLTIVTLAISVISLVYIMG^49^.

### Phylogenetic analysis of complete genome, complete F and HN genes of strains F and R2B

**Figure 1:**
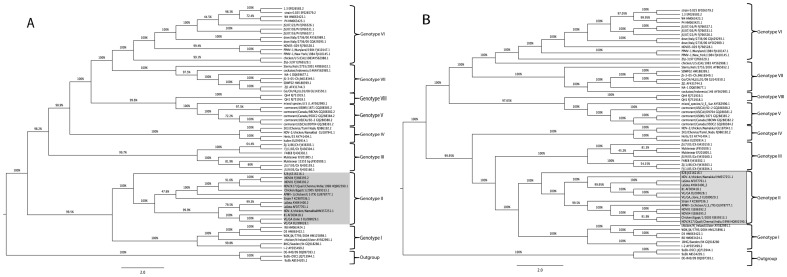
Phylogenetic analysis of complete genome of vaccine strains R2B and F. The whole genome sequence of NDV strain R2B and F was aligned with other NDV strain sequences from GenBank representing different genotypes using Muscle algorithm in MEGA5.0. A. A rooted consensus tree drawn to scale was obtained using Maximum Likelihood method employed with discrete gamma distribution model with evolutionary invariable (GTR+G+I) using MEGA5.0. B. A Bayesian tree analysis of complete genome analysis of Indian vaccine strains. The scale indicates the number of substitutions per site. Posterior probabilities are indicated above the branches.

The commonly used Indian vaccine strains of NDV grouped along with genotype II viruses that also contained viral strains exhibiting different virulence attributes, such as La Sota, B1, VG/GA that are lentogenic, Beaudette C that is mesogenic and Texas GB (TX/GB) being velogenic. The vaccine strain F was more closely related to Beaudette C strain than to La Sota or B1. The vaccine strain R2B branched out separately from the two sub-clusters within genotype II viruses, one cluster involving the lentogenic vaccine strains La Sota, B1, VG/GA and the other with virulent virus strains Beaudette C, Texas GB and Egypt/2005 (Figure 1).

**Figure 2:**
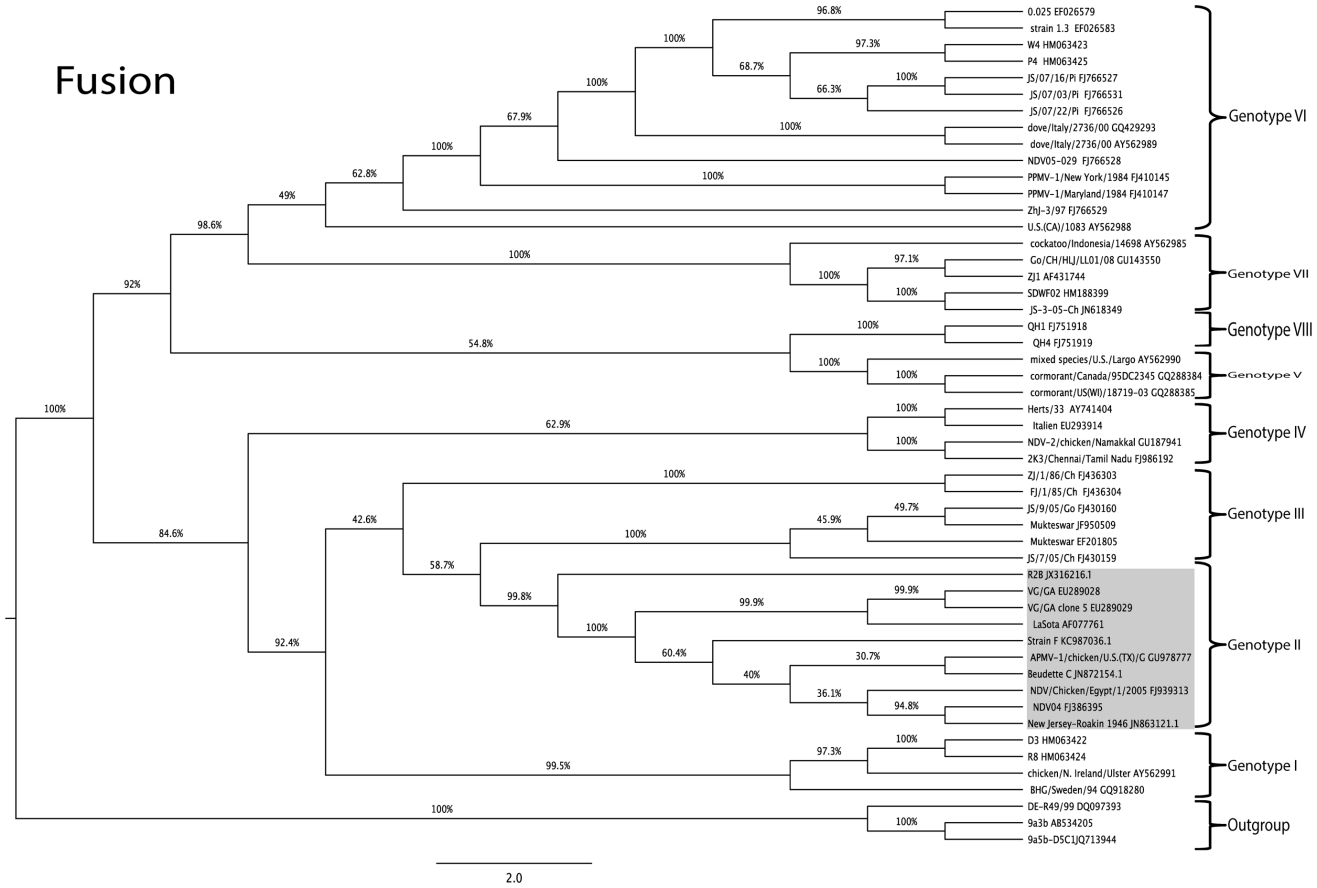
Phylogenetic relationship of vaccine strains R2B and strain F (boxed) based on Fusion protein gene. The phylogenetic tree was drawn using maximum likelihood method employed in MEGA program (Version 5). The percentage of replicate trees in which associate taxa clustered together in the bootstrap test (1000 replicates) is shown next to the branches. Trees are drawn to scale, with branch length in the same units as those of the evolutionary distances used to infer the phylogenetic tree. The evolutionary distances were computed and are in the units of the number of base substitutions per site.

**Figure 3:**
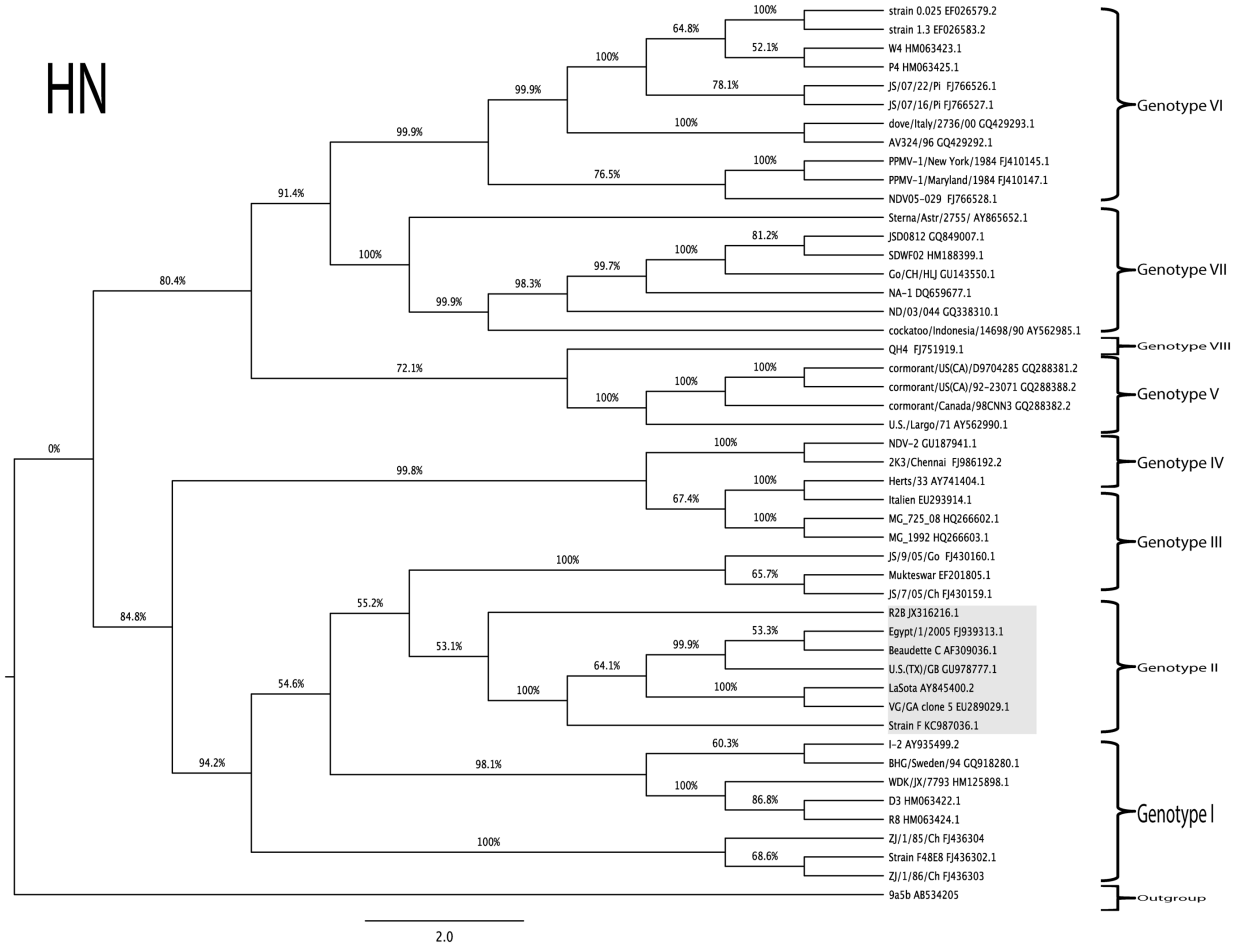
Phylogenetic relationship of vaccine strains R2B and strain F (boxed) based on HN protein gene. The phylogenetic tree was drawn using maximum likelihood method employed in MEGA program (Version 5). The percentage of replicate trees in which associate taxa clustered together in the bootstrap test (1000 replicates) is shown next to the branches. Trees are drawn to scale, with branch length in the same units as those of the evolutionary distances used to infer the phylogenetic tree. The evolutionary distances were computed and are in the units of the number of base substitutions per site.

Further, the topology of the tree did not have any significant differences when the complete fusion and HN genes of the viruses were used for comparison in this study (Figure 2 and Figure 3).

### Pathogenicity

The NDV vaccine strains F and R2B had a MDT of 184 and 63 hrs, ICPI value of 0.1 and 1.45 and IVPI value of 0.0 and 0.55 respectively, confirming the lentogenicity and mesogenicity of the strains.

## Discussion

The backyard poultry sector in India is increasingly being recognized as an important area of intervention for poverty alleviation. Because rearing is based on traditional practices with no focus on veterinary and health sciences, there is high mortality caused by diseases. Newcastle disease is identified as the most fatal disease wiping out entire flocks, severely constraining the growth of this sector. Vaccination is the principal method of controlling the losses caused by ND. For prophylactic use, the lentogenic strains of NDV of chick embryo origin, such as B1, La Sota and F are likely used as vaccines in most countries of the world. In addition, mesogenic strains of NDV, Komarov and R2B are still used extensively in many Asian countries, including India for its very strong immune response evoked in susceptible birds even up to 4 years [26]. The puzzling persistence of virulent NDV in poultry despite intensive vaccination efforts has been a recurrent phenomenon in endemic countries of Asia, Africa and Central America [27], [4]. Almost exclusive predominance of low virulence class I and mesogenic virus of class II [4] suggests that the immune pressure from vaccination may be selecting variant form of virulent NDV.

Both the vaccine strains used in this study have a genome length of 15,186 nts, suggesting that these belong to the ‘early’ (1930–1960) genotypes among the class II ND viruses [4]. This could be further corroborated with their years of isolation being 1950 for strain F in England, and 1945 for strain R2B in India. Phylogenetic analysis of complete genome of these vaccine strains aligned them with class II genotype II viruses, which also harbour some of the commonly used vaccine strains across the world including B1, La Sota, VG/GA and Beaudette C. Interestingly, this genotype also includes the neurotropic virulent chicken/U.S (TX) GB/1948 (TXGB) isolate which was isolated in 1948, and is used in the USA as a challenge virus to show efficacy of ND commercial vaccines before production [5]. Within the genotype II viruses, two distinct separate groups were delineated in which lentogenic strains, such as B1, La Sota, VG/GA formed a group and mesogenic/velogenic strains, such as Beaudette C, TXGB and Egypt/2005 formed another group. The vaccine strain F was related to the mesogenic group of viruses, while R2B strain forming a separate clade branching out from both these groups but within genotype II. This is in concurrence with the earlier findings [28], [29], wherein the FPCS region of the virus was used to study the relationship among different viruses. The topology of the tree and the grouping of Indian vaccine strains did not change when the full-length F and HN genes were compared with the sequences belonging to different genotypes from the GenBank.

Both NDV surface glycoproteins interact during infection of cells [30]. The principal function of paramyxovirus F protein is pH independent cell fusion, following cleavage activation by cellular proteases [31]. All five potential glycosylation sites were conserved among NDV isolates and at least four of them are utilized in the ectodomain of the mature protein [32]. The F glycoprotein of strains R2B and F contains six potential N-linked glycosylation acceptor sites at residues 85, 191, 366, 447, 471, and 541 that are all conserved in other NDV strains. Cysteines are important for disulphide bond formation in the F protein [33], [34]. R2B strain has 13 cysteine residues, whereas strain F carries 12, which is similar to another vaccine strain Ulster.

Virulence of NDV isolates is primarily determined by sequence at the F cleavage site from positions 112 to 116 [35]. The difference in the cleavage site of both the vaccine strains used in this study is indicative of the difference in the virulence attribute of these strains, namely lentogenic for strain F and mesogenic for strain R2B in corroboration with the pathogenicity test data.

An interesting feature, especially with the genome of R2B in relation to the polymerase gene, is that this genome is closely related to the genome of Egypt/2005 which has been designated as a velogenic virus [36]. This further substantiates to the fact that there are other virulence factors such as polymerase gene, which also play a major role in determining the virulence of NDV [21].

With reference to the HN protein, different amino acid sequence lengths of 571, 577, 581 and 616 have been reported for different NDV strains [37]. Analyzing the HN sequences of different NDV strains, a 571 amino acid HN protein, which is the shortest of all, was found in genotypes III–VIII. In this context, the length of HN protein of strain Mukteswar also has 571 amino acids (consisting exclusively of viscerotropic, velogenic strains). Genotype II strains had 6 amino acids extension regardless of whether the strain was lentogenic, mesogenic or velogenic [38]. Strains F and R2B with different virulence attributes have 577 amino acids that concurred with this fact. This is in contradiction to the findings of Zanetti et al. [39] who showed that the NDV pathogenicity increased through serial viral passages by introduction of point mutation at the carboxy terminus region of HN to shorten this protein. However, based on experimental evidence it has been proven that the length of HN protein has no role in NDV pathogenicity [19].

India being an endemic country for NDV, outbreaks of the disease occur every passing year. It has also been recently reported about the persistence of genotype IV strains in India [40]. The results of the study reported herein indicate that the conventional vaccines like strain F and R2B to belong to genotype II. There have been reports suggesting that vaccination against NDV although protects against clinical disease, it fails to protect against virus shedding when challenged with a different genotype virus [41], [42]. Considering these facts, it can be concluded that the commonly used vaccine strains in India need better jurisprudence in its usage as a prophylactic agent.
